# Development of Toxicological Risk Assessment Models for Acute and Chronic Exposure to Pollutants

**DOI:** 10.3390/toxins8090251

**Published:** 2016-08-31

**Authors:** Elke S. Reichwaldt, Daniel Stone, Dani J. Barrington, Som C. Sinang, Anas Ghadouani

**Affiliations:** 1 Aquatic Ecology and Ecosystem Studies, School of Civil, Environmental and Mining Engineering, The University of Western Australia, 35 Stirling Highway, M015, Crawley WA 6009, Western Australia, Australia; elke.reichwaldt@uwa.edu.au (E.S.R.); 20514707@student.uwa.edu.au (D.S.); 2 International Water Centre, Department of Marketing, Monash University, School of Public Health, The University of Queensland, Level 16, 333 Ann Street, Brisbane QLD 4000, Queensland, Australia; dani.barrington@monash.edu; 3 Faculty of Science and Mathematics, Sultan Idris Education University, Tanjong Malim 35900, Perak, Malaysia; somcit@fsmt.upsi.edu.my

**Keywords:** environmental risk assessment, pollutant, cyanobacteria, chronic risk, acute risk, microcystins, probability of exposure

## Abstract

Alert level frameworks advise agencies on a sequence of monitoring and management actions, and are implemented so as to reduce the risk of the public coming into contact with hazardous substances. Their effectiveness relies on the detection of the hazard, but with many systems not receiving any regular monitoring, pollution events often go undetected. We developed toxicological risk assessment models for acute and chronic exposure to pollutants that incorporate the probabilities that the public will come into contact with undetected pollution events, to identify the level of risk a system poses in regards to the pollutant. As a proof of concept, we successfully demonstrated that the models could be applied to determine probabilities of acute and chronic illness types related to recreational activities in waterbodies containing cyanotoxins. Using the acute model, we identified lakes that present a ‘high’ risk to develop Day Away From Work illness, and lakes that present a ‘low’ or ‘medium’ risk to develop First Aid Cases when used for swimming. The developed risk models succeeded in categorising lakes according to their risk level to the public in an objective way. Modelling by how much the probability of public exposure has to decrease to lower the risks to acceptable levels will enable authorities to identify suitable control measures and monitoring strategies. We suggest broadening the application of these models to other contaminants.

## 1. Introduction

Water pollution is a major threat to human health and one of humanity’s biggest challenges [1,2]. One approach to reduce the risk that pollution events pose to the public is the use of alert level frameworks that are based on thresholds for health-harmful pollution levels [3,4]. Their implementation is triggered by the detection of a pollution event. Alert level frameworks differentiate between the seriousness of consequences that arise from a range of exposures, allowing managers to progressively adapt their response by restricting access, passive monitoring or active treatment (e.g., [5,6,7,8,9,10]). Their focus is to facilitate response management in that they serve to identify actions to be put into place to reduce the acute risks posed by the immediate, detected occurrence of pollution. Through these alert level frameworks, agencies are generally well prepared to respond to such discrete pollution events. However, their effectiveness is based on the fact that the pollution is detected and reported in the first place.

With many systems not receiving any regular monitoring, pollution events often go undetected. This potentially puts the public at risk. The probability of being exposed to a hazard that is undetected depends on the monitoring frequency of a system [11], which is governed by the allocation of limited financial resources to agencies and environmental projects [12]. However, any suggestion to increase monitoring efforts is unrealistic as it is not economically viable for most agencies. Instead, understanding and identifying the risk a system poses to the public in regards to a pollutant of interest, assuming that not all pollution levels are detected due to infrequent monitoring would be an important step towards developing meaningful and sustainable management approaches.

The overall objective of this study was to develop toxicological risk assessment models for acute and chronic exposure to pollutants which incorporate the probabilities that the public comes into contact with undetected pollution events. These models can be used to assess the overall risk a system poses to the public with regards to a chosen pollutant and inform authorities on strategic management, including monitoring strategies and implementation of measures such as banning of recreational use to reduce the risk to the public.

We illustrate the development of the models using microcystins, which are toxins produced by aquatic cyanobacteria, as the pollutant of interest. To assess the applicability of the developed models, we then applied them to data collected by Sinang, Reichwaldt and Ghadouani [13], who monitored microcystin concentrations in urban lakes. Microcystins pose a major threat to human health when present in drinking water, recreational waterbodies and in water used for crop irrigation [14,15]. They can cause acute poisoning and, through chronic exposure, liver cancer [5,16,17,18,19,20]. As cyanobacterial bloom frequency and toxin occurrence will likely increase in the future due to climate change and increasing nutrient pollution of waterbodies [21,22,23,24] and because microcystins have been shown to persist in lake sediment and accumulate in the food web [25,26] it is essential to develop management strategies that are informed by risk assessment to ensure the safety of the public. While the models are developed for acute and chronic risk assessment of microcystins they are general and can be adapted to any pollutant by replacing respective pollutant thresholds.

## 2. Framework Development

### 2.1. Development of Conceptual Frameworks for Acute and Chronic Risk Assessments

**Figure 1:**
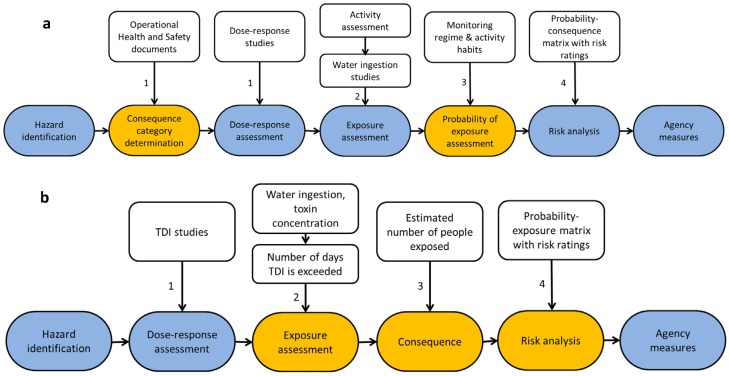
Conceptual framework for (**a**) acute and (**b**) chronic risk assessment models. Blue boxes present steps from previous risk assessment frameworks (adapted from [8]). Orange boxes represent steps that are newly incorporated or adapted within this newly proposed framework to allow an accurate estimation of the risk by assessing the probabilities that humans come into contact with undetected pollution (acute framework) or the probability that chronic illnesses are developed (chronic framework). In addition, to allow agencies to make decisions on the use of water, a step that determines the potential consequences from coming into contact with the pollutant (consequence category determination) is included in both frameworks. Please note that the activity assessment in the acute framework (**a**) incorporates the duration of exposure, the pattern of exposure and the exposure pathway described in [8]. Numbers refer to steps described in the method section.

Two conceptual frameworks, for acute and chronic risk assessment models, respectively, were developed. Existing risk assessment frameworks were adapted to include the probability that the public comes into contact with a pollution source and to allow the assessment of the chronic risk (Figure 1). The existing frameworks have four main steps in common: hazard identification, exposure assessment, dose-response assessment and risk analysis [10]. In the case of cyanobacterial blooms, the hazard may be the presence of cyanotoxins (e.g., microcystin) [8]. The purpose of the second step, the exposure assessment, is to determine the exposure pathways and the duration and dose of exposure. The third step, the dose-response assessment, is used to determine the relationship between the dosage an individual receives and the seriousness of the expected response. In practice, the severity of responses occurs on a spectrum which is difficult to quantify and depends on individual characteristics such as fitness and weight. This, together with categorising risks according to the severity of the consequence will allow objective management of the pollution hazard and has been incorporated into the models developed here (Figure 1a, step 1). The last step, the risk analysis, involves characterising risks to develop risk ratings that help agencies to develop safer practises or help them to decide on the measures that need to be adopted to decrease the risk [27].

These previous frameworks rely on existing data on the range of possible responses of an individual to ingesting and absorbing a given quantity of toxic substance and on the concentration that the substance must have in the water in order to yield each of the possible responses. However, they do not consider two important factors: first, the probability that an individual comes into contact with the pollution event; second, any chronic exposure of an individual due to a regular contact with the water source, because previous frameworks are developed for actual pollution events only. To close these gaps, we incorporated this into existing frameworks (Figure 1a,b).

### 2.2. Development of the Acute Risk Assessment Model

#### 2.2.1. Consequence Category Determination and Derivations of Dose-Response Values for Microcystin-LR (Step 1)

Contact with cyanotoxins includes ingestion, respiration and dermal contact and can result in a number of symptoms and illnesses. For the acute risk assessment model, the spectrum of the severity of responses is simplified by describing distinct consequence categories [28] and determining dosage thresholds for those categories (Table 1). These consequence categories can be adapted by utilities to fit their risk analysis. The dosage thresholds displayed in Table 1 were determined by reviewing guideline values (e.g., [29]) and toxicological studies into the effects of microcystin-LR, the most toxic microcystin (MC) congener.

The least severe consequence of coming into contact with microcystins would be a First Aid Case (Table 1). This can be caused by dermal contact and allergic reactions leading to rashes. A recent laboratory study showed that keratinocytes in skin cultures can be damaged and lose their regenerative capacity for a short period of time when exposed to concentrations of microcystin-LR in excess of 10 µg·MC·L^−1^ [30].

The most severe consequence of exposure to microcystins is death. The acute toxicity lethal dose (LD_50_) of mice for orally administered microcystin-LR was 5 mg·MC·kg^−1^ body weight (b.w.) [18] and this value has been used by the World Health Organization (WHO) to develop tolerable daily intake amounts [5,31]. We adopted this concentration as a dose threshold for this consequence category, applying an uncertainty factor of 1000. Uncertainty factors are applied to provide a margin of safety due to limitations in toxicological studies such as the use of non-human species in these studies, limited data availability or the variability in responses of human individuals [5,31,32,33].

For the Day Away From Work category no concentration thresholds for once-off exposures have previously been established. Therefore, for this category we adopted concentrations derived from chronic exposure experiments [16] that are the basis for the tolerable daily intake (TDI) used by the WHO [5,31], assuming that above these concentrations a person becomes ill. Fawell, James and James [16] determined the No-Observable-Adverse-Effect-Level (NOAEL) in mice as 40 µg·MC·kg^−1^, with degeneration of liver cells occurring at higher concentrations in a study where doses were administered ranging from 40 to 1000 µg·MC·kg^−1^ over a 13 week period. We calculated the dose threshold concentration for the Day Away From Work category (Table 1) by dividing the NOAEL by the uncertainty factor of 1000. Please note that using this concentration as the acute risk threshold is highly conservative as the doses required to cause a specific consequence are likely to be higher when the concentration is administered only once (acute), instead of continuously (chronic).

For the Long Term Injury category the dose threshold concentration was derived from Ito, Kondo and Harada [34], who found that administering 500 µg·MC·kg^−1^ b.w. orally to mice once led to liver injury after 2 hours. As such, we calculated the dose threshold concentration for the Long Term Injury category (Table 1) by dividing this concentration by the uncertainty factor of 1000.

#### 2.2.2. Exposure Assessment and Derivations of Ingestion Values during a Given Activity (Step 2)

The purpose of the exposure assessment (Figure 1a, step 2) is to determine the pathways of microcystin-LR uptake and how much water might be taken in by the body, in order to derive the concentration of microcystin-LR required to result in a certain consequence category. There are two main pathways by which microcystins pose health risks to individuals. Individuals can either come into physical contact, or can ingest water containing microcystins [35]. Unless individuals have an open wound, the toxin is unable to penetrate through the skin, although it may cause irritation resulting in a First Aid Case [30]. For more serious consequences to eventuate the individual must ingest the water. Breathing in water droplets that contain microcystins has also been suggested as a possible ingestion pathway [36]. A lack of reliable data prevented an inclusion of this pathway within this work; however, once, more evidence becomes available, it can be incorporated into the developed models.

Two major scenarios are considered here as they encapsulate the range of circumstances by which individuals may be exposed to cyanobacteria during recreational activities: swimming and other “non-immersive” water sports, such as boating. The concentration of microcystins in the water that is required to produce a certain consequence category while performing a specific activity can be determined as

(1) $$C_{consequence}=\frac{D_{consequence}}{V_{activity}}\times M$$

where $C_{consequence}$ is the concentration of microcystin-LR (µg·L^−1^) in the water to give a specific consequence, $D_{consequence}$ is the dose threshold (µg·kg^−1^) required to give a specific consequence (Table 1), M is the bodyweight (kg) of the individual affected, and $V_{activity}$ is the volume of water (L) expected to be ingested during a given activity. Please note that $C_{consequence}$ is independent of M and $V_{activity}$ for injuries resulting from skin contact and in such case would be equal to $D_{consequence}$.

The volume of water ingested during a specific activity is highly variable between individuals. The calculation of guidelines by the WHO for safe exposure to microcystins in recreational water [31] has used an ingestion volume of 0.2 L·day^−1^ [31] (p. 170). This value seems high compared to values suggested by Schets, Schijven and de Roda Husman [37] who determined that the average amount of water ingested during a swimming event in a lake is 27, 18 and 37 mL for men, women and children, respectively. People participating in ‘non-immersive’ water sports were found to ingest half of the quantity ingested while swimming [38]. Despite the evidence that the ingestion value used by WHO is highly conservative we have adopted them (200 mL for swimming, 100 mL for boating) for our case study for consistency with other risk assessments.

**Figure 2:**
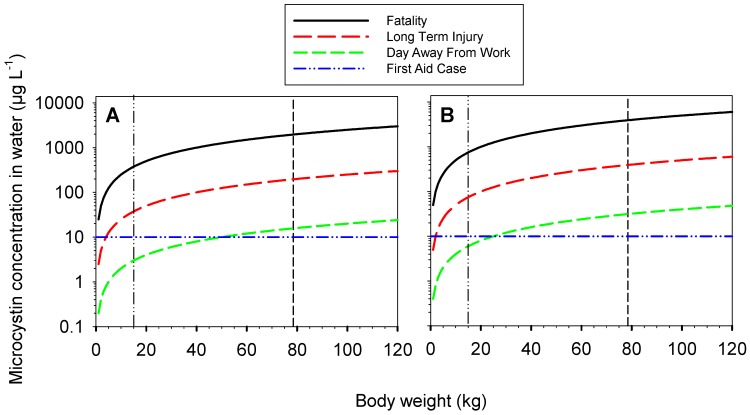
Concentrations (μg·L^−1^) of microcystin-LR that are expected to cause different consequence categories (i.e., First Aid, Day Away From Work, Long Term Injury, Fatality) as a function of body weight for (**A**) swimming and (**B**) boating. Short broken lines indicate a typical Australian adult (78.5 kg) [39], dash-dotted lines an Australian five year old child with the lowest decile weight of 15 kg [40]. The ingestion volumes used are 200 mL (swimming) and 100 mL (boating).

Using these ingestion volumes, concentration thresholds (*C_consequence_*; Equation (1)) that lead to specific consequences can be calculated for the two scenarios (i.e., swimming, non-immersive boating) as a function of body weight (Figure 2). This can be used to identify likely consequences during microcystin pollution for individuals of different body weight and activity.

#### 2.2.3. Probability of Exposure Assessment, Informed by Monitoring Regime and Activity Habits (Step 3)

If all blooms were to be detected with adequate measures put in place subsequently and people adhering to these measures, the probability of a person to come into contact with cyanotoxins (=probability of exposure; *P_exposure_*) would be zero. In the real world it is neither possible nor sensible to monitor every waterbody continuously due to financial constraints and, as such, it is unlikely that all blooms are detected. Consequently, some blooms are not identified in time to prevent exposure. An assessment of the probability of exposure therefore has to take into account the probability that a bloom will go unnoticed together with the probability that an individual also comes into contact with this bloom. As such, the monitoring regime and the activity habits of people have to be taken into account (Figure 1a, step 3). Barrington, Ghadouani, Sinang and Ivey [11] developed a risked-based framework that allows water utilities to design water quality monitoring regimes based on the probability of bloom detection. Within this framework, they developed an equation to calculate the probability (*To*, Tolerance) that an agency’s ongoing monitoring regime will fail to identify a hazard (= microcystin pollution) when one is present:

(2) $$To=\left(1-\frac{1-HF}{1-\left(HF\times MF\right)}\right)$$

where *To* is the probability that the regime will not detect microcystins (i.e., *To* = 0.2 suggests that the agency will not detect microcystins in 20% of cases where microcystins are actually occurring), *MF* is the monitoring frequency (per week), and *HF* is the frequency of the hazard (e.g., specific concentration of microcystins, *C_consequence_,* exceeded; per week) actually occurring, determined from historical monitoring data [11]. This equation can be rearranged if an agency has defined a tolerance threshold, so as to inform them of the ideal monitoring frequency (*MF*) they should undertake.

Where consistent monitoring has created a historical dataset, the hazard frequency (*HF*) can be calculated as a product of the proportion of times the hazard has been detected to exceed a defined category threshold concentration (e.g., the concentration that will cause a Day Away From Work) and the *MF* of the water body (Equation (3)).

(3) $$HF=\frac{Number\;of\;Times\;Consequence\;Threshold\;Exceeded}{Total\;Number\;of\;Data\;Points}\times MF$$

Equation (2) assumes that if the hazardous event occurs and is not identified by monitoring that it will result in some individual being exposed to the hazard. However, this might not be the case as it depends on factors such as the popularity of a system to be used by the public. By including the probability that an individual will come into contact with or ingest sufficient water during the time when microcystins are present ($P_{exposure}$) in Equation (2), the contact frequency (*CF*; week^−1^) can be calculated (Equation (4)), which is the frequency with which a person comes into contact with a defined hazard.

(4) $$CF=P_{exposure}\times \left(1-\frac{1-HF}{1-\left(HF\times MF\right)}\right)$$

$P_{exposure}$ can be determined by intensive monitoring of peoples’ activity habits. For example, a lake for boating that is close to a city might be used almost every day$\;(P_{exposure}=1)$ by people, while a lake further away from a city may only be used on weekends $(P_{exposure}=0.29\;,\;$i.e., two out of seven days per week). The probability that a contact is occurring (CP; weeks) is then calculated as

(5) $$CP=\frac{1}{CF}$$

#### 2.2.4. Risk Analysis and Risk Ratings (Step 4)

Risk analysis is done by identifying risk ratings (i.e., very low risk to very high risk) [27], which are applied worldwide in the form of probability-consequence matrices [41]. The probability-consequence matrix in Table 2 uses the contact frequency (*CP*) and the dose thresholds (*C_consequence_*) for each consequence category, each of which is objectively calculated. This probability-consequence matrix should be adapted by agencies in line with their existing risk management policies.

### 2.3. Development of the Chronic Risk Assessment Model

#### 2.3.1. Derivations of Tolerable Daily Intake (TDI) Values for the Chronic Dose-Response Assessment (Step 1)

Chronic exposure to microcystin-LR manifests as long-term liver damage and the promotion of tumour growth [6]. The chronic dose response to toxins is estimated based on the number of days per year the Tolerable Daily Intake (TDI) is exceeded [42]. TDI is the maximum daily dosage of a chemical which during an entire lifetime poses no risk and can be exceeded for short periods of time without chronic risk [32]. TDIs are derived from a No-Observable-Adverse-Effect-Level (NOAEL) established from laboratory experiments and by dividing the NOAEL by an uncertainty factor of 1000.

Few studies have investigated the long term effects of microcystin-LR. WHO has adopted TDI guideline values for microcystin-LR of 0.04 µg·MC·kg^−1^ b.w. [31,32] based on the study by Fawell et al. [16] described earlier and we have adopted the use of this value for the purpose of this case study.

#### 2.3.2. Derivation of Values for Exposure Assessment (Days) Based on Probability Ratings for the Development of a Chronic Illness and Water Ingestion Volumes (Step 2)

The Tolerable Daily Intake (TDI) describes the amount of toxin that, when ingested on a daily basis, will not result in any harm [35]. However, there is no guideline which indicates the number of days the TDI must be exceeded to result in injury. In the absence of studies that test the dose-response to frequency of exceeding the TDI, ratings have been developed for the probability that a chronic illness will develop based on the number of days the TDI is exceeded (Table 3), which is estimated from the duration of chronic toxicological studies [16,18,43,44].

The purpose of the exposure assessment (Figure 1b, step 2) is to provide an estimate of the number of days an individual’s TDI will be exceeded to identify probability ratings that a chronic illness will develop (Table 3). The exposure assessment is based on two components: monitoring data which characterise the historical microcystin-LR concentration over time and the probability of ingesting water based on the type of activity. Equation (6) has been developed from these two components to estimate the number of days per annum (*N*) that an individual will exceed their TDI of microcystin-LR:

(6) $$N=n\times \overset{n}{\underset{i\;=\;0}{\sum }}P{\left(contam\right)}_{i}\times P{\left(ingest\right)}_{i}$$

where *P(contam)_i_* is the probability of water being contaminated with microcystin-LR of a concentration level *i*, equivalent to its historical frequency obtained from historical monitoring data; *n* is the maximum number of days an individual is exposed to the water source per year; and *P(ingest)_i_* is the probability of an individual ingesting the quantity of water required to exceed their TDI during a certain activity, which can be estimated based on probability distributions extracted from Schets, Schijven and de Roda Husman [37] using the cumulative distribution function of a gamma distribution [45] (see Table S1).

#### 2.3.3. Estimating the Number of People Exposed to Inform the Consequence (Step 3)

Given the likely narrow range of consequences caused by chronic exposure to microcystins (i.e., liver cancer, fatality) a typical consequence-probability matrix, such as the one developed for the acute risk model (Table 2), would only have one consequence category. Therefore, it is proposed that the number of people, who are exposed to the water be used in lieu of consequence, as consequence can be considered a function of the number of people injured.

#### 2.3.4. Risk Analysis and Risk Ratings (Step 4)

Using the amount of days the TDI is exceeded (Table 3) and the number of people exposed in lieu of consequence, a chronic probability—exposure matrix (Table 4) can be developed to determine the chronic risk rating posed by a specific water body for a given activity. Note that the risk rating for one individual is identical to the probability rating in Table 3.

### 2.4. Proof of Concept: Assessing the Risk of Using Lakes for Recreational Swimming

To test the applicability of the developed acute and chronic models we applied them to weekly microcystin concentration data from five urban lakes collected by Sinang, Reichwaldt and Ghadouani [13] assuming the public use them for a range of activities. More details on the sampling regime and the lakes can be found in Sinang, Reichwaldt and Ghadouani [13] and microcystin concentrations for each lake are summarised in Table S2.

## 3. Results

### 3.1. Acute Risk Assessment Model

To evaluate the acute risk of microcystin poisoning for the public, the risk rating of the most severe consequence that the public could expect to have from a lake was identified, and the probabilities of exposure (*P_exposure_*) which would result in lower risk ratings were modelled. Therefore, this model could be used by agencies to help identify required control measures to reduce the risk to an acceptable level.

#### 3.1.1. Determination of Most Severe Consequence (Step 1)

The historical dataset of microcystin-LR concentrations in the five urban lakes (Table S2) was compared with the consequence-concentration thresholds (*C_consequence_*; Equation (1)) for an adult of Australian median weight (*M*) (78.5 kg) during a swimming activity (*V_activity_* = 200 mL of water ingested). Using the *D_consequence_* values for each consequence (Table 1), *C_consequence_* values were calculated to be 15.7 µg·MC·L^−1^ (Day Away From Work), 196.3 µg·MC·L^−1^ (Long Term Injury) and 1963 µg·MC·L^−1^ (Fatality). The consequence threshold for a First Aid Case is independent of body weight and is 10 µg·MC·L^−1^. Table 5 summarises the highest concentrations of microcystin-LR measured in each lake and the corresponding consequences.

#### 3.1.2. Incorporation of Monitoring Regime (MF) and Probability of Exposure (P_exposure_) to Identify Contact Frequency (CF) and Contact Probability (CP) (Step 2)

Table 5 summarises *MF*, *HF*, *CF* and *CP* for each lake. *HF* for the most severe consequence expected was determined using Equation (3), with *MF* being the number of sampling dates divided by the total number of weeks (Table S2). The contact frequencies (*CF*; Equation (4)) and contact probabilities (*CP*; Equation (5)) for each consequence were then calculated using two different *P_exposure_*. The first risk assessment was conducted assuming that no controls are applied by authorities. This presumes that no warning signs are present and people use this lake daily for swimming (*P_exposure_* = 1). A second risk assessment was calculated assuming that this lake is used less frequently for swimming (50%) either because it is less popular or because some measure has been put in place that reduces the probability of the public to swim in this lake (*P_exposure_* = 0.5).

#### 3.1.3. Risk Analysis: Using the Most Severe Consequence Categories Identified and the Contact Probability (CP) to Determine Risk Rating (Step 3)

The risk rating is then characterised (Table 6) using the semi-quantitative probability-consequence matrix (Table 2). Without applying any controls, and with the lake being used by the public daily, lakes 2 and 4 both pose a high risk to obtain Day Away From Work injuries from undetected blooms, while the most severe consequences for lakes 1, 3, 5 are First Aid Cases and the risks that these occur are low (lake 1) and medium (lakes 3, 5). Reducing the probability of coming into contact with the microcystins to 50% (*P_exposure_* = 0.5) reduces the risk of the most severe consequence identified for each lake only in lakes 1, 3, 5.

#### 3.1.4. Modelling the Required Probability of Exposure *P_exposure_* to Achieve Desired Risk Rating Categories (Step 4)

**Figure 3:**
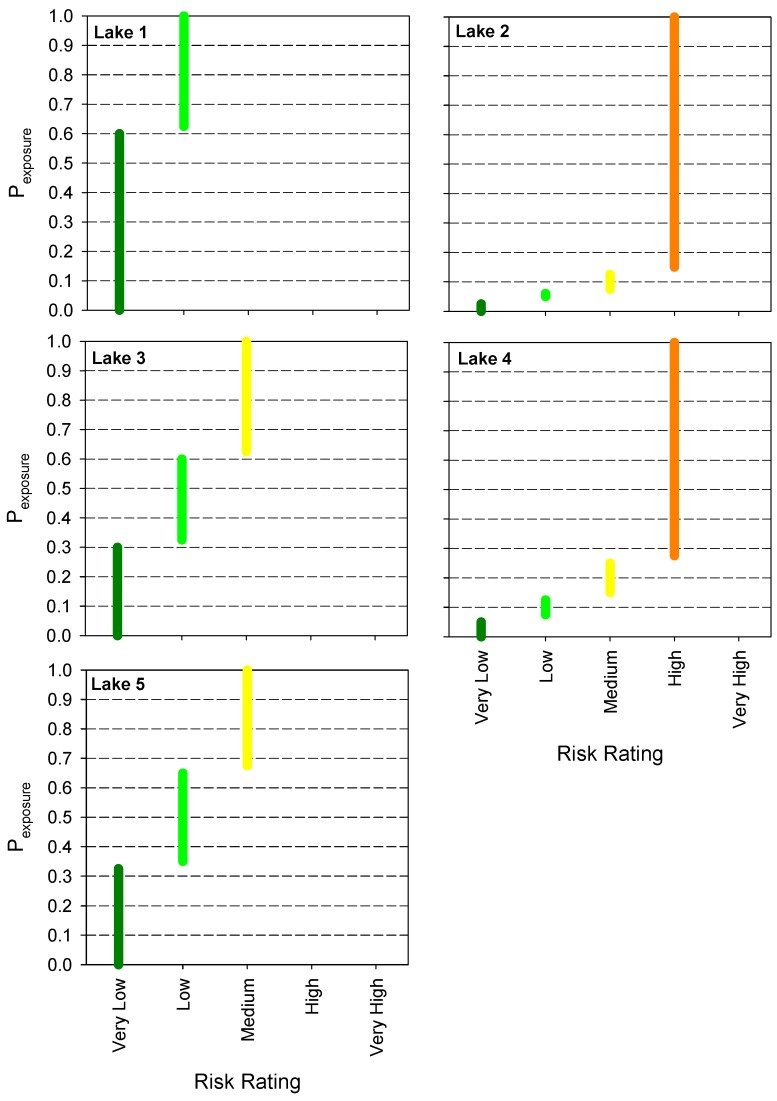
The probability of exposure (*P_exposure_*) to be chosen to achieve desired risk ratings for the five lakes, calculated for the most severe consequence for each lake, i.e., First Aid Case for lakes 1, 3, 5 and Day Away From Work Cases for lakes 2, 4 (Table 5). *P_exposure_* can be adjusted by introducing controls to prevent access of contact with the hazard.

Using the approach detailed in the previous step 3, the contact probability (*CP*) for each probability of exposure (*P_exposure_*), ranging from 0 to 1, is calculated using Equation (5), and is then converted into risk ratings using Table 2. This step enables agencies to determine the required probability of exposure (*P_exposure_*) to achieve a desired risk category for each lake. Plotting *P_exposure_* against the risk categories reveals that there are two categories of lakes (Figure 3): the first category encompasses lakes 1, 3, 5 for which a reduction of the most severe consequence to a lower risk level can be achieved relatively easily by putting controls into place that reduce the probability of exposure to around 0.6. For the other two lakes, *P_exposure_* has to be decreased to 0.1 (lake 2) and 0.25 (lake 4), indicating that stronger measures are needed to prevent access of the public.

### 3.2. Chronic Risk Assessment Model

To identify the chronic risk ratings for the lakes three steps are required. We here demonstrate detailed calculations for lake 2 as an example (Table 7), with the risk ratings for all lakes given in Table 8.

#### 3.2.1. Assessing the Volume of Water Required to Exceed the TDI (Step 1)

For each concentration data point, the volume of water required to be ingested by an adult of median Australian Weight (78.5 kg) to exceed the TDI (3.14 µg·MC = 78.5 kg × 0.04 µg·MC·kg^−1^) was determined (Table 7, column B) by dividing the TDI by the microcystin concentration in the water and multiplying it by 1000.

#### 3.2.2. Estimating the Number of Days the TDI Is Exceeded (Step 2)

The probability of ingesting (*P(ingest)_i_*) the previously determined volume of water (Table 7, column B) is identified using the probability distribution (Supplement Materials Table S1). Next, the number of days that each sample represents for the monitoring period has to be quantified. In the absence of daily microcystin concentrations, it is assumed that each data point was representative of the continuous microcystin-LR concentration in the waterbody between two monitoring days and this was converted into days per year (Table 7, column D). This step would not be necessary if the microcystin concentration was quantified daily throughout the year. For this case study, two scenarios were modelled to estimate the numbers of days that the TDI was exceeded: a 56-day scenario (swimming once per week) and a 252-day scenario (swimming every day for 9 months in the year). An estimate of the number of days that the TDI was exceeded for an individual (*N*) was then calculated using Equation (6). The results indicate that a swimmer using lake 2 once per week is likely to exceed the TDI for 9 days throughout a year, while this increases to 41 days if they swim on 252 days (every weekday) within one year.

Following the same approach, the number of days that a swimmer exceeded their TDI (*N*) can be calculated for all other lakes (lakes 1 and 3–5) (Table 8). The results indicate that lakes 2 and 4 are the only other lakes where swimmers exceed their TDI on some days throughout the year (Table 8).

#### 3.2.3. Estimating Usage and Determining Risk Rating (Step 3)

Using the previously developed probability rating (Table 4), the risk that an individual develops a chronic illness due to swimming was ‘very low’ in all lakes under both scenarios with the exception of lake 2, where the probability increased to ‘low’ if an individual swims daily for 9 months per year (252-day scenario) (Table 8). Assuming that in a Mediterranean climate the public would use lakes for swimming regularly during the warmer months, and in the absence of usage data, we estimated that 100 people are using each lake. Consequently, the overall risk rating for the public (consisting of 100 people per lake) was ‘low’ for all lakes under both scenarios (56-day and 252-day scenario), except for lake 2 where the risk was medium for the 252-day scenario (Table 8).

## 4. Discussion

The models developed here serve to assess the potential acute and chronic risks that systems pose to the health of the public with respect to a pollutant of interest. In comparison with existing frameworks which are emergency responses to single occurring pollution events [31], our models integrate the probabilities that the public comes into contact with undetected pollution events and as such present an important risk management tool for agencies.

Applying the acute and chronic models to historical monitoring data demonstrated its potential to determine the expected health outcomes of recreational activities related to waterbodies containing microcystin-LR. As the frameworks are based on toxicological studies and risk management guidelines, it delivers an objective risk assessment using the information that is available for a pollutant. Integrating these frameworks into management practises can support agencies and councils in identifying lakes that pose a high threat to people and can further serve as a decision making tool towards identifying the appropriate control measures to lower the risk. Our model identifies the risk to people, if a system is not monitored at all. The results suggest that if a lake is used by the public only infrequently for swimming and if a low chronic risk is acceptable, then having no monitoring or very infrequent monitoring might be appropriate.

Our study highlights the significant impact of controls on acute risk ratings. Controls for protecting the public from pollutants, including cyanobacterial blooms in lakes are usually in the form of warning or regulatory signs [9] or alert warnings in local newspapers. In addition, risk education has been identified as an important way to increase awareness and change behaviour [46] and could help increase the number of people heeding the warning signs. However, these controls do not avoid the human fragility of oversight [47] and research indicates that even the best signs are not obeyed by 100% of those seeing them [48]. Guidelines for standard signs for water safety are available [9] but their effectiveness in having people obey them depends on motivational and social circumstances highlighting the complexity of developing effective controls to reduce the exposure with the hazard [48]. The risk rating of a lake can also be decreased by reducing the number of pollution events. This can be achieved by preventing contamination from outside sources including fining polluters or by adopting lake management techniques that are known to decrease the presence of the pollutant within the lake (e.g., [49,50]).

The accuracy and outcomes of the models developed here strongly depend on the availability of toxicological studies that identified relevant concentration levels of interest and on the risk appetite of the authorities, which will affect the risk ratings. Due to the relatively limited number of toxicological studies into microcystin-LR and studies into the ingestion of water for risk assessment from different activities, there are several significant sources of uncertainty in the results in our case study, including: the uncertainty in the toxicological dose dependent response to obtain defined consequences during exposure, and the uncertainty in the amount of water ingested per activity per day. As acute incidents may be triggered by a single, high-exposure event, it is necessary to estimate the maximum volume of water that an individual might ingest, rather than use estimates of the amount of water regularly ingested [31,35,37,51,52]. In addition, the accuracy of the risk calculated using these frameworks strongly depends on the availability of true long-term data of a system. Ideal are direct measurements of the pollutant at high frequencies, in particular when pollutant concentrations are highly variable over short time periods due to pollutant production and degradation [13,53]. In practise, such data sets are rare. In the case for managing cyanobacteria, waterbodies are often analysed monthly or fortnightly and the limited resources allow only the quantification of cell numbers of potentially toxic cyanobacterial species. Converting those cell numbers into toxin concentrations adds another uncertainty that greatly affects the outcome of the framework. These sources of uncertainty are addressed in guidelines and risk assessment frameworks, including the ones presented here by incorporating high uncertainty factors, assuming worst case scenarios in relation to ingestion volumes, and factors to convert cell counts to toxin concentrations (e.g., [6]). As such, the risk ratings identified here can be considered as highly conservative.

The reliability and accuracy of risk models depends strongly on the quality of the data on critical doses and the identification of potential short term (acute) and long-term effects on humans [54]. For microcystins, as seen in our case study, the absence of a toxicological study into the time-dependent response of mice to doses exceeding the TDI (chronic) and the lack of a study unifying the dose-dependent acute consequences to exposure resulted in the need to extract data from multiple studies to characterise these responses. Further, while the ingestion of water during swimming has been well studied [37,51,52], we could only find one study for people participating in non-immersive water sports other than swimming [38]. Despite this, we successfully demonstrated that the models performed well and were able to identify different risk levels for the lakes, depending on the recreational activity level. Using the models for other pollutants, for which better knowledge of doses and acute and long term effects exists, will increase their accuracy.

The frameworks developed here can be adapted to any pollutant that shows a dose-dependent response to exposure (e.g., *E. coli* [55]), including gaseous pollutants like hydrogen sulfide (H_2_S) and greenhouse gases [56], because only the dose dependent response components of the risk assessment frameworks are specific to the toxicology of microcystin-LR. Its application might be especially important in the wastewater sector due to an increased reuse of wastewater for various purposes. Further, wastewater contains more pollutants than lakes, many of which are biological hazards. With several agencies acknowledging that the potential risk from chemicals is lower than from water borne biological hazards (cyanobacteria) [9,32], we suggest to further test its applicability for a wider range of contaminants, water bodies and activities.

## 5. Conclusions

The proposed risk models succeeded in characterising risk in a way which is both objective and relevant to decision makers. The acute and chronic models were both able to characterise the risk and differentiate between proposed acceptable and unacceptable risks, and can be used to identify by how much the probability of exposure has to be decreased to lower the risk to an acceptable level. This will enable management authorities to identify appropriate control measures to be put in place, such as warning or regulatory signs, alert warnings in local newspapers, and increased monitoring and fining of polluters. We suggest broadening the application of these models by investigating their application to other contaminants and to waterbodies with multiple constituents of concern.

The accuracy of the results of the risk assessment models depend upon precise input values from toxicological studies of pollutants (here: microcystin-LR) and studies into ingestion volumes during specified activities. This uncertainty is intensified by the absence of any toxicological study that covers the entire spectrum of injuries possible through acute microcystin-LR exposure. Therefore in order to conduct a dose-dependent response assessment, dosage values must be extracted from different studies with varying methodologies, increasing the level of uncertainty in the assessment. We therefore call for more standardised toxicological studies on the health effects of contaminates, including cyanobacterial toxins, which would improve the accuracy of the results delivered by the developed models.

## Figures and Tables

**Table 1:** Summary of the consequence categories (after [28]) and consequence threshold dosages derived from toxicological studies for the acute risk assessment of microcystin-LR. Due to inter-species and intra-species variability, there is significant uncertainty in assuming that the dose responses determined in toxicological studies of microcystin-LR using mice and pigs are similar in humans. To adjust for uncertainty, the dose thresholds in this table have been calculated by dividing literature-derived dose thresholds that were obtained from non-human studies (all except [30]) by an uncertainty factor of 1000 [5,32,33]. The dose threshold for fatality is based on the LD_50_.

Consequence Category	Definition	Example Symptoms	Dose Threshold (*D_consequence_*)	References
First Aid	A superficial injury requiring first aid treatment	Skin conditions; allergic reactions	10 µg·MC·L^−1^ (dermal contact)	[30]
Day Away from Work	A temporary injury that requires rest away from work	Respiratory problems, gastroenteritis	0.04 µg·MC·kg^−1^ b.w.	[16]
Long Term Injury	An incident where an individual receives a permanent injury	Liver failure, tumour growth	0.5 µg·MC·kg^−1^ b.w.	[34]
Fatality	A death that directly results from acute exposure	Death through acute toxicity	5 µg·MC·kg^−1^ b.w.	[18]

b.w. = body weight.

**Table 2:** Acute probability—consequence matrix based on contact probability (*CP*) of an event (probability rating) and expected consequence categories showing risk ratings (very low–very high).

	Consequence	First Aid	Day Away From Work	Long Term Injury	Fatality
Contact Probability (*CP*)					
Once per 10 years or less		Very Low	Very Low	Low	Low
Once per 5–10 years		Very Low	Very Low	Low	Medium
Once per 2–5 years		Very Low	Low	Medium	High
Once per 1–2 years		Low	Medium	High	Very High
Once per 0.5–1 years		Medium	High	Very high	Very High
More than twice per year		High	High	Very High	Very High

**Table 3:** Suggested probability rating for the development of a chronic illness based on the number of days TDI is exceeded per annum.

Days TDI Is Exceeded (*N*)	Probability Rating	Justification
0–39	Very Low	No chronic injury resulted from this exposure time [18].
40–99	Low	One study [43] suggests that this number of days can result in injury.
90–139	Medium	Three studies [16,18,43] suggest that this number of days can result in injury.
140–179	High	An intake above the TDI for over one third of human life poses higher probability of incident occurring.
≥180	Very High	One study indicates that even concentrations of microcystins below the TDI can lead to adverse health effects when administered daily for 6 months [44].

**Table 4:** Chronic probability—exposure matrix showing risk ratings (very low–very high) based on the categories identifying the probability to develop chronic illness (days of TDI exceeded per year; Table 3) and the number of people exposed.

	People Exposed	1	10	100	1000
TDI Exceeded (Days·Year^−1^)					
0–39		Very Low	Low	Low	Medium
40–89		Low	Low	Medium	High
90–139		Medium	Medium	High	Very High
140–179		High	High	Very High	Very High
≥180		Very High	Very High	Very High	Very High

**Table 5:** Summary of highest microcystin-LR concentrations (c(MC) in μg·L^−1^) recorded in the five urban lakes, the most severe expected consequence resulting from this concentration when the lake is used for swimming, and the calculated monitoring frequency (*MF*; week^−1^), hazard frequency (*HF*; week^−1^), contact frequency (*CF*; week^−1^) and contact probability (*CP*; week) of the most severe consequence events occurring calculated for each lake. The expected consequences are derived from comparison with the concentration thresholds for the four consequence categories (*C_consequence_*), namely 10.0 µg·MC·L^−1^ (First Aid Case), 15.7 µg·MC·L^−1^ (Day Away From Work), 196.3 µg·MC·L^−1^ (Long Term Injury) and 1963 µg·MC·L^−1^ (Fatality). *P_exposure_* = 1 indicates that *CF* values are calculated with no control mechanisms from authorities in place and the lake being used for swimming daily. *P_exposure_* = 0.5 represents a scenario where the probability of the public to come into contact with the hazard is reduced to 50%.

Lake	Highest c(MC)	Expected Consequence	*MF*	*HF*	*P_exposure_* = 1		*P_exposure_* = 0.5	
					*CF*	*CP*	*CF*	*CP*
1	10	First Aid Case	0.43	0.027	0.016	64	0.008	129
2	122	Day Away From Work	0.48	0.238	0.141	7	0.070	14
3	11	First Aid Case	0.44	0.056	0.032	32	0.016	63
4	118	Day Away From Work	0.61	0.167	0.072	14	0.036	28
5	13	First Aid Case	0.50	0.056	0.029	35	0.014	70

**Table 6:** Summary of the most severe consequence detected and the Contact Probability (*CP*) used to determine the Risk Rating for each lake.

Lake	Consequence	*P_exposure_* = 1		*P_exposure_* = 0.5	
		*CP*	Risk Rating	*CP*	Risk Rating
1	First Aid Case	1 in 64 weeks	Low	1 in 129 weeks	Very Low
2	Day Away From Work	1 in 7 weeks	High	1 in 14 weeks	High
3	First Aid Case	1 in 32 weeks	Medium	1 in 63 weeks	Low
4	Day Away From Work	1 in 14 weeks	High	1 in 28 weeks	High
5	First Aid Case	1 in 35 weeks	Medium	1 in 70 weeks	Low

**Table 7:** Calculation of the number of days per year (*N*; Equation (6)) that an individual will exceed their TDI of microcystin-LR intake when swimming in lake 2. *P(contam)_i_* is the probability of water being contaminated with microcystin-LR of the concentration level *i* (µg·MC·L^−1^); *P(ingest)_i_* is the probability of an individual ingesting the quantity of water required to exceed their TDI during a certain activity and can be taken from Table S1. Days equivalent is the number of days per monitoring duration each sample represents; *n* is the maximum number of days an individual is exposed to the water source per year (52-day, and 252-day scenario; see text).

Date	A	B	C	D	E	F
	*i*	Volume to Exceed TDI (mL)	*P(ingest)_i_*	Days Equivalent	*P(contam)_i_*	C × E
10 November 2008	0.1	22429	0	36.5	0.100	0
8 December 2008	0.2	15700	0	36.5	0.100	0
12 January 2009	0.5	6826	0	36.5	0.100	0
9 February 2009	13.4	234	0	36.5	0.100	0
16 February 2009	122.1	26	0.646	36.5	0.100	0.065
23 February 2009	122.3	26	0.646	36.5	0.100	0.065
9 March 2009	83.2	38	0.316	36.5	0.100	0.0316
16 March 2009	21.1	149	0	36.5	0.100	0
23 March 2009	37.0	85	0.003	36.5	0.100	0.0003
6 April 2009	7.2	434	0	36.5	0.100	0
					**Sum**	**0.161**
				***N;*** ***n* = 56 (scenario 1)**		**9**
				***N;*** ***n* = 252 (scenario 2)**		**41**

**Table 8:** Chronic Risk Assessment results including the probability rating that a chronic illness will develop and the overall risk rating for five urban lakes that are used for swimming by 1 person (individual) or 100 people (group) once per week (days of exposure, *n* = 56; scenario 1), or 1 person or 100 people swimming daily for 9 months in a year (days of exposure, *n* = 252; scenario 2). *N* is the number of days per year that an individual will exceed their TDI.

Lake	*N*		Probability Rating (Individual)		Risk Rating (Group)	
	Scenario 1	Scenario 2	Scenario 1	Scenario 2	Scenario 1	Scenario 2
1	0	0	Very Low	Very Low	Low	Low
2	9	41	Very Low	Low	Low	Medium
3	0	0	Very Low	Very Low	Low	Low
4	2	11	Very Low	Very Low	Low	Low
5	0	0	Very Low	Very Low	Low	Low

## Supplementary Materials

The following are available online at www.mdpi.com/2072-6651/8/9/251/s1, Table S1: Probability of ingesting a given volume of water during swimming and “non-immersive” water sports, such as boating (adapted from [37]), calculated by using the cumulative distribution function of a gamma distribution [45], Table S2: Microcystin concentration (µg·MC·L^−1^) in five urban lakes (from [13]).
