# Supplementary material for: Development of Toxicological Risk Assessment Models for Acute and Chronic Exposure to Pollutants
Source: Toxins (Basel). 2016 Aug 31;8(9):251. doi: 10.3390/toxins8090251 (PMC5037477; doi:10.3390/toxins8090251)
Supplement: Supplementary file 1 [file toxins-08-00251-s001.pdf]

# Supplemental Material: Development of Toxicological Risk Assessment Models for Acute and Chronic Exposure to Pollutants

**Elke S. Reichwaldt, Daniel Stone, Dani J. Barrington, Som C. Sinang and Anas Ghadouani**

**Table S1.** Probability of ingesting a given volume of water during swimming and "non-immersive" water sports, such as boating (adapted from [37]) calculated by using the cumulative distribution function of a gamma distribution [45].

| Volume (mL) | Swimming Probability | Boating Probability |
|---|---|---|
| 0 | 1 | 1 |
| 2 | 0.99999 | 0.99971 |
| 4 | 0.99971 | 0.99335 |
| 6 | 0.9981 | 0.96639 |
| 8 | 0.99335 | 0.90771 |
| 10 | 0.98337 | 0.81762 |
| 12 | 0.96639 | 0.70574 |
| 14 | 0.94127 | 0.58525 |
| 16 | 0.90771 | 0.46805 |
| 18 | 0.86614 | 0.36246 |
| 20 | 0.81762 | 0.27282 |
| 22 | 0.7636 | 0.20027 |
| 24 | 0.70574 | 0.1438 |
| 26 | 0.64576 | 0.10126 |
| 28 | 0.58525 | 0.07007 |
| 30 | 0.52562 | 0.04774 |
| 32 | 0.46805 | 0.03207 |
| 34 | 0.41344 | 0.02128 |
| 36 | 0.36246 | 0.01396 |
| 38 | 0.31551 | 0.00906 |
| 40 | 0.27282 | 0.00583 |
| 42 | 0.23444 | 0.00372 |
| 44 | 0.20027 | 0.00235 |
| 46 | 0.17015 | 0.00148 |
| 48 | 0.1438 | 0.00092 |
| 50 | 0.12095 | 0.00057 |
| 52 | 0.10126 | 0.00035 |
| 54 | 0.0844 | 0.00022 |
| 56 | 0.07007 | 0.00013 |
| 58 | 0.05794 | 0.00008 |
| 60 | 0.04774 | 0.00005 |
| 62 | 0.03919 | 0.00003 |
| 64 | 0.03207 | 0.00002 |
| 66 | 0.02616 | 0.00001 |
| 68 | 0.02128 | 0.00001 |
| 70 | 0.01726 | 0 |
| 72 | 0.01396 | - |
| 74 | 0.01126 | - |
| 76 | 0.00906 | - |
| 78 | 0.00728 | - |

**Table 1.** *Cont.*

| Volume (mL) | Swimming Probability | Boating Probability |
|---|---|---|
| 80 | 0.00583 | - |
| 82 | 0.00466 | - |
| 84 | 0.00372 | - |
| 86 | 0.00296 | - |
| 88 | 0.00235 | - |
| 90 | 0.00186 | - |
| 92 | 0.00148 | - |
| 94 | 0.00117 | - |
| 96 | 0.00092 | - |
| 98 | 0.00073 | - |
| 100 | 0.00057 | - |
| 102 | 0.00045 | - |
| 104 | 0.00035 | - |
| 106 | 0.00028 | - |
| 108 | 0.00022 | - |
| 110 | 0.00017 | - |
| 112 | 0.00013 | - |
| 114 | 0.0001 | - |
| 116 | 0.00008 | - |
| 118 | 0.00006 | - |
| 120 | 0.00005 | - |
| 122 | 0.00004 | - |
| 124 | 0.00003 | - |
| 126 | 0.00002 | - |
| 128 | 0.00002 | - |
| 130 | 0.00001 | - |
| 132 | 0.00001 | - |
| 134 | 0.00001 | - |
| 136 | 0.00001 | - |

**Table S2.** Microcystin concentration (μg·MC·L$^{-1}$) in five urban lakes (from [13]).

| Date | Lake 1 | Lake 2 | Lake 3 | Lake 4 | Lake 5 |
|---|---|---|---|---|---|
| 3 November 2008 | 10.16 | - | - | - | - |
| 10 November 2008 | - | 0.14 | 3.51 | 13.58 | 5.07 |
| 17 November 2008 | - | - | - | 5.72 | - |
| 1 December 2008 | - | - | - | 12.48 | 2.15 |
| 8 December 2008 | 8.82 | 0.2 | 0.05 | 16.13 | 7.59 |
| 12 January 2009 | - | 0.46 | 0.14 | 25.63 | 6.49 |
| 19 January 2009 | 3.69 | - | 7.14 | 1.14 | 12.96 |
| 26 January 2009 | 3.1 | - | 11.27 | 1.77 | 2.88 |
| 2 February 2009 | - | - | 2.84 | 1.05 | 2.61 |
| 9 February 2009 | 0.92 | 13.42 | 8.55 | 7.43 | 2.97 |
| 16 February 2009 | - | 122.08 | - | 3.74 | - |
| 23 February 2009 | 1.08 | 122.34 | - | 23.63 | - |
| 9 March 2009 | 1.82 | 83.2 | - | 3.94 | - |
| 16 March 2009 | - | 21.11 | 2.7 | - | 0.46 |
| 23 March 2009 | 8.98 | 36.98 | - | 5.39 | - |
| 6 April 2009 | 2.23 | 7.23 | - | 3.06 | - |
| 13 April 2009 | 2.04 | - | - | 17.32 | - |
| 27 April 2009 | 3.51 | - | - | 6.15 | - |
| 18 May 2009 | 6.25 | - | - | 118.16 | - |
| 25 May 2009 | - | - | - | 86.07 | - |
| 1 June 2009 | 3.33 | - | - | 13.07 | - |
| 22 June 2009 | 1.95 | - | - | 4.48 | - |
| 6 July 2009 | 3.52 | - | - | 3.52 | - |
| 20 July 2009 | 3.4 | - | - | 1.54 | - |
| Number of sampling dates | 16 | 10 | 8 | 22 | 9 |
| Number of weeks | 37 | 21 | 18 | 36 | 18 |
